# Stochastic modeling of oligodendrocyte generation in cell culture: model validation with time-lapse data

**DOI:** 10.1186/1742-4682-3-21

**Published:** 2006-05-17

**Authors:** Ollivier Hyrien, Ibro Ambeskovic, Margot Mayer-Proschel, Mark Noble, Andrei Yakovlev

**Affiliations:** 1 Department of Biostatistics and Computational Biology, University of Rochester, 601 Elmwood Avenue, Rochester, New York 14642, USA; 2 Department of Biomedical Genetics, University of Rochester, 601 Elmwood Avenue, Rochester, New York 14642, USA

## Abstract

**Background:**

The purpose of this paper is two-fold. The first objective is to validate the assumptions behind a stochastic model developed earlier by these authors to describe oligodendrocyte generation in cell culture. The second is to generate time-lapse data that may help biomathematicians to build stochastic models of cell proliferation and differentiation under other experimental scenarios.

**Results:**

Using time-lapse video recording it is possible to follow the individual evolutions of different cells within each clone. This experimental technique is very laborious and cannot replace model-based quantitative inference from clonal data. However, it is unrivalled in validating the structure of a stochastic model intended to describe cell proliferation and differentiation at the clonal level. In this paper, such data are reported and analyzed for oligodendrocyte precursor cells cultured *in vitro*.

**Conclusion:**

The results strongly support the validity of the most basic assumptions underpinning the previously proposed model of oligodendrocyte development in cell culture. However, there are some discrepancies; the most important is that the contribution of progenitor cell death to cell kinetics in this experimental system has been underestimated.

## Background

The theory of branching stochastic processes has proved a powerful tool for cell kinetics in general and for analyzing clonal growth of cultured cells in particular. The ongoing development of mathematical aspects of this theory is frequently stimulated by or directed towards applied problems. A comprehensive account of the theory and some biological applications are given in books by Harris [1], Sevastyanov [2], Mode [3], Athreya and Ney [4], Jagers [5], Assmussen and Hering [6], Yakovlev and Yanev [7], Guttorp [8], Kimmel and Axelrod [9] and Haccou et al. [10].

Since the choice of a particular model is frequently determined by its tractability, the Bellman-Harris branching process and its modifications have been traditionally considered as a fairly general framework for cell kinetics studies. The multi-type version of this process is defined as follows. Let , *i,k *= 1, ..., *K *be the number of cells of the *k*^th ^type at time *t *given that the clonal growth starts with a single (initiator) cell of type *i *at time *t *= 0. The vector **Z**^(i)^(t) = (, ..., ) is said to be a Bellman-Harris branching stochastic process with *K *types of cells if the following conditions are met. Each cell of type *k*, 1 ≤ *k *≤ *K*, transforms into *j*_1_, ..., *j*_*K*_, daughter cells of types 1, ..., *K*, respectively, with probability *p*_*k*_(*j*_1_, ..., *j*_*K*_). The time to transformation is a non-negative random variable (r.v.) with cumulative distribution function (c.d.f.) *F*_*k*_(.). The usual independence assumptions are adopted.

The problem of quantitative inference from clonal data on cell development in tissue culture has been addressed in our publications [11-21]. These papers employ a multi-type Bellman-Harris branching process to model the proliferation of oligodendrocyte/type-2 astrocyte progenitor cells and their transformation into terminally differentiated oligodendrocytes. This model is widely applicable to other *in vitro *cell systems. The precursor cell that gives rise directly to oligodendrocytes was first discovered by Raff, Miller and Noble in 1983 [22], when it was named as an oligodendrocyte/type-2 astrocyte (O-2A) progenitor for the two cell types it could generate *in vitro*. This cell is also known as an oligodendrocyte precursor cell (OPC), and will be referred to henceforth as an O-2A/OPC. Such cells appear to be present in various regions of the perinatal rat CNS, and cells with similar properties also have been isolated from the human CNS [23].

The O2A/OPC-oligodendrocyte lineage has provided a remarkably useful system for studying general problems in cellular and developmental biology. In the context of our present studies, three advantages of this lineage are that it is possible to analyze progenitor cells grown at the clonal level, that progenitor cells and oligodendrocytes can be readily distinguished visually, and that the generation of oligodendrocytes is associated with exit from the cell cycle. In the culture system we use in our experiments, the dividing O-2A/OPCs only make either more progenitor cells or oligodendrocytes; no other branching in the process of their development is possible. This makes it possible to conduct well-controlled experiments that generate quantitative information on cell division and differentiation at the clonal level and at the level of individual cells.

The earlier proposed model was designed to describe the development of cell clones derived from O-2A/OPCs under *in vitro *conditions. Cells of this type are partially committed to further differentiation into oligodendrocytes but they retain the ability to proliferate. It is believed that the main function of progenitor cells *in vivo *is to provide a quick proliferative response to an increased demand for cells in the population. Terminally differentiated oligodendrocytes represent a final cell type; they are responsible for maintaining tissue-specific functions and they do not divide under normal conditions. Both cell types are susceptible, in variable degrees, to death.

A substantial amount of new biological knowledge has emerged from applications of our model to experimental data, with a particular focus on understanding the regulation of differentiation at the clonal level. As all differentiation processes require that cells make a decision between differentiating and not differentiating, it is important to understand how this process is controlled at the level of the individual dividing precursor cell. Early studies had indicated that individual O-2A/OPCs would divide a limited number of times before all clonally related cells differentiated synchronously and symmetrically under the control of a cell-intrinsic biological clock. Subsequent biological studies showed that the cell-intrinsic regulator of differentiation promoted asymmetric and asynchronous differentiation among clonally related cells unless promoters of oligodendrocyte generation were present. It was only through our modeling studies, however, that the popular clock model of oligodendrocyte generation *in vitro *was disproved by testing a more general (hierarchical) model against experimental data [11,15].

In the earliest version of our model [11,15], it was assumed that the initial population of progenitors is a mixture of subpopulations with different numbers of "critical" cycles. In each of these subpopulations the probability of division is 1 until the critical number is reached and drops sharply to a fixed value *p *< 0.5 afterwards. The number of critical cycles is not directly observed, and one can only verify this basic assumption by fitting the model to experimental data on the evolution (over time) of clones consisting of two distinct types of cells. However, if one considers the whole population of cells, there is a more gradual decline in the division probability from 1 to *p*, suggesting that an alternative model is also plausible, in which there is a single population of progenitor cells with a gradually decreasing division probability [17]. While both models are in almost equally good agreement with clonal data, the latter model has a more parsimonious structure, which is also perfectly consistent with the time-lapse data to be reported in the present paper.

The basic stochastic model of proliferation and differentiation of O-2A/OPCs was based on the following assumptions:

*A*1. The process starts with a single progenitor cell of type 1 at time 0.

*A*2. After completion of its mitotic cycle, every progenitor cell of type *l *≥ 1 either divides to produce two new progenitor cells of age 0 and type *l *+ 1 with probability *p*_*l*_, or transforms into a differentiated cell of type *l *= 0 (oligodendrocyte) with probability 1 - *p*_*l*_.

*A*3. The time to division of a progenitor cell of type *l *≥ 1 is a non-negative r.v. *T*_*l*,1 _with c.d.f. *F*_1_(*x*), while the time to differentiation of a progenitor cell of type *l *≥ 1 is a non-negative r.v. *T*_*l*,2 _with c.d.f. *F*_2_(*x*).

*A*4. Differentiated oligodendrocytes neither divide nor differentiate further, but they may die; their lifespan *T*_0 _has c.d.f. *L*(*x*) = Pr(T_0 _≤ *x*).

*A*5. Whenever counts of dead oligodendrocytes are utilized for estimation purposes, the model needs to be extended further to include the following assumption: every dead oligodendrocyte disappears (disintegrates) from the field of observation after a random lapse of time *T*_-1 _distributed in accordance with c.d.f. *H*(*x*) = Pr(*T*_-1 _≤ *x*). The time to the disintegration event is expected to be quite long, as there are no macrophages present in the culture to clear away cell debris.

*A*6. The cells do not migrate out of the field of observation.

*A*7. Of the two cell types, oligodendrocytes appear to be more susceptible to death. Therefore, it was assumed that progenitor cells do not die during the period of observation.

*A*8. The assumption of independence of cell evolutions is adopted. This assumption is critical for making the mathematical treatment of the resultant branching stochastic process tractable.

The probabilities *p*_*l *_can be described by an arbitrary function of the mitotic cycle label *l *that satisfies the natural constraints: 0 ≤ *p*_*l *_≤ 1 for all *l *≥ 1. In [17], these probabilities are specified as *p*_*l *_= min{*p *+ *qr*^*l*^, 1}, where *p*, *q *and *r *are free positive parameters with *p *representing the limiting probability of division of progenitor cells as the number of cycles tends to infinity. In our analysis of the time lapse data in the next section we proceeded from this choice as well. All the distributions introduced above were specified by a two-parameter family of gamma distributions, which is the most popular choice in cell kinetics studies [7].

Assumption A3 was introduced in [19,20] to allow the mitotic cycle duration and the time to differentiation to follow dissimilar distribution functions. The authors proceeded from the following line of reasoning. In the classical Bellman-Harris process, either the event of division or the event of differentiation is allowed to occur upon completion of the mitotic cycle. Let the r.v.s *X *and *Y *represent the time to division and the time to differentiation, respectively. Then the postulates of the Bellman-Harris process imply that the joint distribution of *X *and *Y *is singular along the diagonal *X *= *Y*. A natural alternative is to assume that the r.v.s *X *and *Y *have dissimilar continuous distributions. This alternative is biologically plausible because the proliferation and differentiation of cells involve different molecular mechanisms. The analysis of clonal growth of cultured O-2A/OPCs has corroborated this hypothesis [19,20], and the time-lapse data presented in the next section provide additional evidence in favor of its validity.

In [18], the mitotic cycle duration and the time to differentiation of O-2A/OPCs were assumed to follow the same distribution, that is, F_1_(*x*) = F_2_(*x*) for all *x*, but we allowed the distribution of the time to division and differentiation of initiator cells to be potentially different from that of cells in subsequent generations. Our time-lapse data provide the opportunity to look more closely at variations in the mitotic cycle duration across cell generations and their consistency with this basic model assumption. The design of our previous studies generated cell counts in independent cell clones at different times after plating. We also used longitudinal data on cell counts produced by observations of the same cell clone at different time points [20]. However, much more information can be extracted from data yielded by time-lapse video recording of individual cell evolutions, and we take advantage of this experimental technique to verify the most basic elements of the earlier proposed model.

## Results and discussion

This study is designed to validate the most basic assumptions behind our model of oligodendrocyte development in cell culture. In what follows, we describe our experimental findings in the context of the model presented in Section 1. Each element of the model structure is discussed separately.

### Mitotic cycle

**Figure 1:**
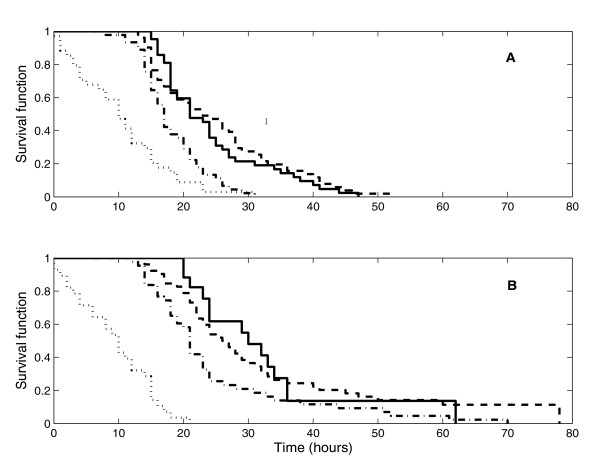
Kaplan-Meier survival curves for the mitotic cycle time across generations. Generation 1 – dotted line, Generation 2 – dash-dotted line, Generation 3 – dashed line, Generation 4 – solid line. Top panel presents data without thyroid hormone; bottom panel shows data with thyroid hormone in the culture medium.

We estimated the distributions of the mitotic cycle duration (MCD) for each generation of progenitor cells. The corresponding Kaplan-Meier estimates are shown on Figure 1. They suggest that the MCD becomes larger as the number of divisions undergone by a progenitor cell increases. However, the log-rank test does not declare these differences to be statistically significant in all pairwise comparisons of the MCD distributions for different generations starting with Generation 3. The fact that the MCD distribution in Generation 1 is distinct from those for other generations is consistent with our previous clonal analyses [18]. The most plausible explanation for this phenomenon is that the initiator progenitor cells sampled *in vivo *are already actively proliferating and, therefore, it is the residual time needed to complete their current mitotic cycle that one observes in cell culture. The second mitotic cycle of the progenitor cells also tends to be shorter than subsequent cycles in both experimental settings (with and without thyroid hormone) but no explanation for this tendency can be offered at present.

The mean MCDs averaged over the generations were estimated as 27.86 hours (standard error (SE) = 0.7 hours) and 22.13 hours (SE = 1.59 hours) in the presence and absence of thyroid hormone, respectively. These estimates are in close agreement with those obtained from clonal data in our past studies [11-17,20]. However, they are different from those reported in [18,19]. This discrepancy is attributable to dissimilar activities of the cytokine PDGF-AA in the culture medium [18]. The effect of thyroid hormone on the MCD distribution is statistically significant (*p *< 0.0001).

**Figure 2:**
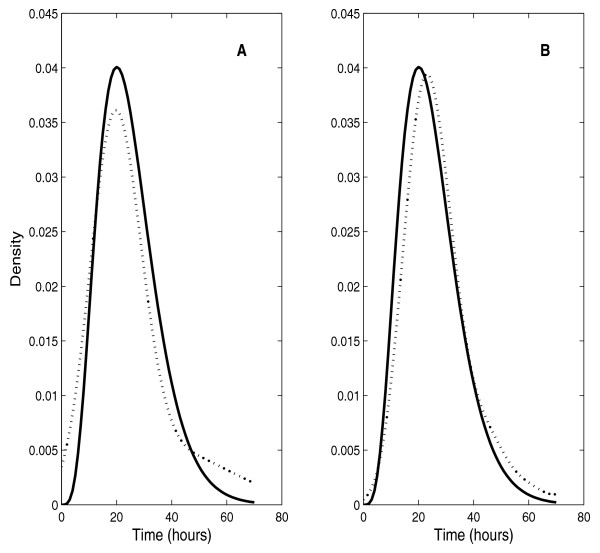
(A) The only case where the null hypothesis is rejected when a gamma distribution density is fitted to observed times to mitotic division; (B) An example where the null hypothesis is not rejected when a gamma distribution density is fitted to observed times to division.

We designed a parametric bootstrap goodness-of-fit test based on the Kolmogorov-Smirnov statistic to test the shape of the MCD distribution. Our study was limited to Generations 1–3 because censoring (by other events such as cell differentiation and death) is too heavy in later generations. A two-parameter gamma distribution provided a good fit for all generations in the absence of thyroid hormone and for Generations 1 and 3 in the presence of thyroid hormone. The only exception was the second generation in the presence of thyroid hormone. In the latter (worst) case, the theoretical gamma distribution and its empirical estimate (kernel estimate with a Gaussian kernel) still coincide quite closely (Figure 2A) so we see no immediate need to replace this approximation with a more flexible parametric family of distributions. For comparison, Figure 2B shows another example where the goodness-of-fit hypothesis was not rejected by the statistical test.

### Probabilities of division, death and differentiation

The probabilities (rates) of death and differentiation increase with generation while the probability of division shows the opposite trend. Notice that the rates of death and differentiation are per cell. The death rate for O-2A/OPCs increases from 0.23 in Generation 2 to 0.57 in Generation 7 in the absence of thyroid hormone and from 0.05 in Generation 2 to 0.11 in Generation 5 in its presence. Therefore, the survival rate of O-2A/OPCs increases in the presence of thyroid hormone. The probability of differentiation increases from 0.07 in Generation 2 to 0.21 in Generation 7 in the absence of thyroid hormone and from 0.18 in Generation 2 to 0.72 in Generation 5 in its presence. This is consistent with the effect of thyroid hormone inferred from our previous analyses of clonal data.

**Figure 3:**
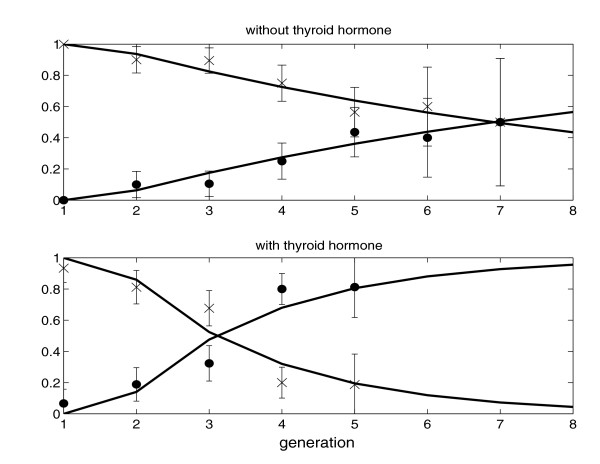
Conditional (given that the cell does not die before the event of interest) probabilities of division (×) and differentiation (circles) of O-2A/OPCs with (lower panel) and without (upper panel) thyroid hormone. The solid lines correspond to the fitted probabilities of division and differentiation, and each error bar indicates two standard errors for the empirical proportion.

Figure 3 shows the estimated conditional probability of division, given that the cell does not die before division or differentiation, as a function of the number of generations. In [17], we used the function *p*_*l *_= min{*p *+ *qr*^*l*^, 1}, *l *≥ 1, to approximate this probability. The same function was used to fit the data in Figure 3 by the non-linear least squares method. Because of conditioning on the event of cell survival, the probability of differentiation equals 1 - *p*_*l*_. It is clear from Figure 3 that the approximation works well.

### Time to differentiation

The overall mean time to differentiation (averaged over the generations) is 31.6 hours (SE = 1.6 hours) for O-2A/OPCs cultured in the presence of thyroid hormone and 31.8 hours (SE = 1.59 hours) in its absence. The time-lapse data confirm that the time to division and the time to differentiation have dissimilar distributions, a conjecture we made earlier from the results of clonal data analysis. The distribution of the differentiation time does not vary significantly across generations (*p *> 0.28). The addition of thyroid hormone has no effect on this distribution.

### Time to death

The overall mean time to death of O-2A/OPCs (averaged over the generations) is equal to 28.1 hours (SE = 2.48 hours) and 19.7 hours (SE = 1.17 hours) with and without thyroid hormone, respectively. These values are very close to the mean mitotic cycle durations recorded in the corresponding experimental settings. The distribution of the time to death for O-2A/OPCs does not vary significantly across generations (*p *> 0.08). Addition of thyroid hormone extends the time to death for O-2A/OPCs (*p *< 0.0018), which is consonant with its positive effect on cell survival.

The presence of thyroid hormone extends the life-time of oligodendrocytes (*p *< 0.0001) as well. The mean time to death of an oligodendrocyte is 19.7 hours in the absence of thyroid hormone but 78.0 hours in its presence. As far as oligodendrocytes are concerned, the estimated overall mean time to death tends to be smaller than our estimates reported in [18] because of the effect of data censoring caused by a limited period of observation [29]. The time to death of oligodendrocytes was not significantly different across generations no matter whether the cells were cultured with or without thyroid hormone (*p *= 0.3 and *p *= 0.27).

### Correlations

We computed correlation coefficients between the times to division for all sister cells and for the corresponding mother-daughter correlations. Because the cells pertaining to the first and second generations had significantly shorter mitotic cycles than those in subsequent generations, we included only the third and later generations in this analysis. The sample correlation coefficients are shown in Table 1. It is clear that the mother-daughter type of correlation is irrelevant to this cell lineage. However, there is a tangible positive correlation between the mitotic cycles of sister cells. Both observations are consistent with the data reported by Powell [24] for bacteria.

**Table 1:** Sample correlation coefficients and their asscciated *p*-values.

correlation type	mother-daughter	*p*-value	sister-sister	*p*-value
without thyroid hormone	0.06	0.6	0.62	<0.0001
with thyroid hormone	0.19	0.5	0.49	0.028

One should expect the mean number of cells not to be affected by this type of correlation, while the variance can only be larger than that in the independent case [1,25]. This was confirmed by our simulation of a population of dividing cells obeying the postulates of the bifurcating autoregressive process. This process [26] reduces to the Bellman-Harris branching process when sister cells have uncorrelated MCD. In this study, we assumed that the logarithms of mitotic cycle times for sister cells have bivariate normal distributions with equal means (25 hours) and equal variances (40 hours), and a fixed positive correlation coefficient denoted by ρ. The bivariate log-normal distribution was chosen as a convenient parametric family for modeling correlations between random variables, while keeping the positivity constraint on cell cycle lengths. The choice of this distribution (instead of the traditional gamma distribution) is of little consequence to the net results of the study. Table 2 displays the standard deviation of the number of cells in this process for ρ = 0 (independent case) and ρ = 0.5, the latter being a reasonable value in accordance with Table 1.

**Table 2:** The standard deviation of a binary splitting Bellman-Harris branching process (no correlation) and the corresponding bifurcating autoregressive process (sister-sister correlation).

Time (days)	3	4	5	6	7	8	9	10	11
ρ = 0.0	0.02	0.3	0.7	0.5	1.1	1.2	1.7	2.3	3.0
ρ = 0.5	0.02	0.3	0.8	0.6	1.3	1.4	2.1	2.7	3.5

The standard deviations of the bifurcating autoregressive process with correlations among sister cells, and the Bellman-Harris process without correlations among sister cells, were estimated from 50000 simulated runs of each process. The observed effect of correlations among sister cells on the standard deviation of the number of cells is rather weak (Table 2). In terms of parameter estimation, this effect translates into a change in the mean MCD of less than 1.5% and a change in the standard deviation of the MCD of less than 3.4%.

Some extensions of the Bellman-Harris branching process have been proposed to allow for dependences between cellular attributes across generations. For example, the bifurcating autoregressive process [26] is designed to model sister-sister and mother-daughter correlations in terms of the MCD. It should be noted that this model describes populations of cells that could divide but neither die nor differentiate. Further improvements of the model and associated methods of statistical inference are being pursued [27]. To the best of our knowledge, the utility of the bifurcating autoregressive process and its various extensions have so far been considered only in the context of time-lapse data. This is not surprising because such data provide abundant information on individual cell evolutions and allow the necessary correlations to be estimated directly.

The situation is not the same when modeling cell development at the clonal (population) level. Except for a few special examples, all stochastic models in cell population kinetics, Markovian or otherwise, disallow for interactions between individual cell evolutions. The same applies indiscriminately to all other stochastic models of discrete entities introduced in mathematical biology, from stochastic models of carcinogenesis or infectious diseases to applications of stochastic processes in ecology and demography. There seems to be no viable alternative to the assumption of independence in all such models as long as they are intended to describe the events of interest at the population level so that their underlying stochastic processes are only partially observed. The main reason for this claim is that stochastic dependencies, such as correlations among sister cells, are basically unobservable at the cell population level and this is exactly the point at which the issue of non-identifiability becomes insurmountable. This, however, does not apply to functional dependencies that may manifest themselves in dynamics of the expected values a typical example is a density dependence such that the net proliferation rate slows down when a set point is reached. A functional dependency of this type may still be identifiable if its structure is parsimonious enough. It should also be noted that, except in some very special cases, branching processes with stochastically dependent cell evolutions are mathematically intractable and we are unaware of a single publication presenting a sufficiently general framework for such processes within which the requisite basic formulae have been derived. Computer simulations with all their inherent problems are the only option in such cases.

The aforesaid, however, does not diminish the usefulness of branching stochastic processes in biological applications. All indirect quantitative inferences from real biological data are conditional on the validity of the assumed model. In other words, we interpret the results of data analysis in terms of model parameters as if all the adopted premises were absolutely valid. In this sense, the assumption of independent evolutions is no different from any other constraint on model structure. It is commonplace to say that all models are wrong but some of them are useful. However, this truism imparts very precisely the essence of mathematical modeling and its place in natural sciences.

On the other hand, biomathematicians should do the best they can to make a mathematical model as realistic as possible, subject to certain constraints on its tractability and identifiability. While alternative variants of a given model most typically emerge when it is in conflict with experimental data, the quest for generality is always warranted in model building. From this perspective, our estimates of sister-sister correlations and the associated simulation study are of practical significance because they show that the observed level of positive correlation among sister cells has only a small effect on the standard deviation of the number of cells at any instant. In accordance with theoretical considerations, this correlation does not affect the expected values at all. These observations provide a rationale for using the method of moments for estimation purposes because, when based only on the mean values and standard deviations, this method appears to be well guarded against correlations between sister cells.

### Other interesting observations

We noticed two unusual events. The first is where the two daughters do not separate fully from each other following division from a mother. For a brief time it looks as if they will separate (1–3 h), but a cytoplasmic bridge between them persists so that eventually they pull back together. This event may be due to spindle dysfunction of the same general kind that leads to tetraploidy in cell culture.

The second event is where the two daughters separate but after a brief period (3–5 h) they track back to each other and appear to merge again into one cell. This type of behavior seems to bear similarities to the reversible incomplete cell separation induced by Cdk1 inhibitors that has recently been reported for primary mammalian cells [28]. In that case, the incomplete separation seems to be a consequence of failure of chromatin segregation. As our cells are cultured in serum-free defined medium in the absence of any chemical Ckd1 inhibitors it is unlikely that these observations are related, although the phenotypic behavior seems very similar.

## Conclusion

This study strongly supports the validity of the assumptions introduced in Section 1. However, it also indicates that the death of progenitor cells is an important element to be incorporated into the model. Before our time-lapse experiments were conducted, we used to believe that the death of O-2A/OPCs was negligible. This belief was based on the results of scoring dead progenitor cells in clonal experiments, which is far less accurate than time-lapse video recording. This experimental evidence was the reason why we did not incorporate the death of O-2A/OPCs into the model.

Previous clonal studies also suggested that the death of oligodendrocytes normally begins on day 7 after plating and its rate increases with time. However, our time-lapse experiments indicate that death begins earlier than we originally thought. While this earlier and more pronounced oligodendrocyte death may be attributable to subtle differences in the growth conditions used in these differing experimental sets, due attention should be given to this discrepancy in future studies.

The time-lapse experiments reported in this paper provide quantitative insight into the correlation structure of realizations of the underlying branching process. Virtually no correlation was observed between the mitotic times of mother and daughter cells. In contrast, the correlation between sister cells is positive and quite high. Among the statistical techniques available for estimating numerical parameters from partially observed branching stochastic processes, moment-based techniques such as the pseudo-maximum likelihood, the least squares, the generalized method of moments or the quasi-likelihood estimators are methods of choice. It follows from our simulation study that the variance of the number of cells appears to be insensitive to the sister-sister correlation of this magnitude, thereby suggesting that the method of moments, as long as it is based on the first two moments, is robust to possible violations of the independence assumption.

The analysis of time-lapse observations reported here suggests certain improvements in the earlier proposed stochastic model of oligodendrocyte generation *in vitro*. This issue invites special investigation and will be addressed in future publications. We hope that many investigators will benefit from the data presented in their efforts to develop useful stochastic models for quantitative analysis of other cell lineages.

## Methods

### 1. Experimental protocol

Oligodendrocyte progenitor cells were isolated from optic nerves of 6 days old rat pups using standard isolation protocols as described in [29] and seeded at a density of 20 k per T-25 flask in DMEM SATO- [30] with 10 ng/ml PDGF-aa. Prior to the start of imaging, 24 h later, the cells were treated with either thyroid hormone T3/T4 (1:1000) to promote oligodendrocyte differentiation [31] or the vehicle (10 mM NaOH). They were then brightfield-imaged on a Nikon TE300 inverted scope equipped with a heated and motorized stage, an atmosphere regulator, and shutter control. The motors controlling the stage and the shutter control were connected to a central control unit, which was in turn connected to a PowerMac G4 computer running IPLab 3.6 software. Using the software, (*x,y,z*) coordinates of 36 fields were recorded and each field was sequentially imaged every 15 min for 138 hours. Once the imaging process was completed, the images were assembled into QuickTime movies using the IPLab software. For analysis, 30 clones were analyzed per experimental condition (60 clones were thus recorded in total), and the time to five kinds of events was recorded for each cell within a clone: division, differentiation, death, exit from the field of view, and the event of censoring due to a limited period of observation. The data were then summarized using clonal trees, where a tree would start with a single cell (a "clone") and would branch out into its progeny, and their fate over time was noted.

### 2. Statistical methods

Most of the data generated by time-lapse experiments are represented by time-to-event observations. A special feature of such data is the presence of censoring effects that need to be accommodated in the statistical inference using methods of survival analysis [32]. The Kaplan-Meier estimator was used to estimate the cumulative time-to-event distribution functions and the corresponding hazard rates. The log-rank test was applied for two-sample comparisons in the presence of right-hand censoring. Since the numbers of observations per generation were not large, we designed a Monte Carlo version of the Kolmogorov test to assess the goodness-of-fit of the gamma distribution chosen to model the MCD distribution. The parameters of the gamma distribution were estimated by the method of maximum likelihood. The test proceeded by first generating bootstrap samples from the fitted gamma distribution. Then the Kolmogorov test statistic was computed for each simulated sample, as well as for the actual sample. The decision rule was similar to the one described in [33].

## Authors' contributions

All the authors contributed equally to this paper. M.M-P. and M.N. were responsible for the biological aspects of this work, including the time-lapse video recording experiments. I.A. conducted the experiments. O.H. and A.Y. were responsible for all aspects of data analysis.
